# The “End of the Season” Illness: Its Sometime Cause and a Case

**Published:** 1906-11

**Authors:** Woodbridge Hall Birchmore

**Affiliations:** Brooklyn, N. Y.


### The "End of the Season" Illness:

### Its Sometime Cause aud a Case.

BY WOODBRIDGE HALL BIRCHMORE, M. D., Brooklyn, N. Y.

AS a consequence of one of the constantly recurring contradictions of all the seeming probabilities of life, "The wise summer maiden, and her wiser yet mamma," who have run every sort of risk during the vacation season, appearing practically immunised to all forms of contagion, are singularly prone to fall ill immediately after they shall have returned to their homes, from the various "places of refuge" at the seaside and among the mountains. Their splendid health during the season has passed into a proverb; and not infrequently we hear words spoken, with intention far from kind, setting forth the excellent health of the invalid away from home. This proposition, that persons who are known by their friends as never well when at home appear always in the best of health, both in body and mind, when absent from its petty cares, is so often demonstrated, that the phrase, "I am as well as an invalid away from home," has passed into a proverb, used by would-be wits to express the very extremity of light heartedness and the most heedless possible enjoyment of the hour.

But it really appears to me that the most interesting part of the picture is passed unnoticed by part of those who one would suppose should see all the details, and those who are in a way conscious of the facts, while appreciating that the conduct of these people is certainly a menace to their health, and the consequences of this misconduct are a mystery, accept the whole strange group of facts as part of life's unexplained phenomena, as if it were not the duty of all interested in the public health to understand the mystery and countervail the menace.

There is also a group who are conscious of both the mystery and the menace, as is shown by the comments which they make from time to time, but while they declare that "Science" should have probed the menace and have shown its power for mischief great or small, and have made the mystery to be easily understood, they are content to see the mystery and the menace remain mystery and menace still.

At every summer watering place we meet these mysterious cases and day after day physicians spend hours in the company of these people who are "Invalids,—Devils at home,—but Angels when away." We find women fair and ever fairer, young and ever younger, and we wonder at the rashness displayed by all of them in every form of pleasure. They appear to defy every rule of right living, to ignore every caution the physicians may have given them, to commit excesses in the plain defiance of all the teachings of sense and reason, and yet seem to go unwhipped of that justice which is a Nemesis for their brothers. Away from home, at the seaside or among the hills, in the cool grotto of some nymph's retreat or wandering along the shimmering sands, these fair matrons and winsome maids are insensible to fear; for them sickness has no threatening hand, Death quite forgotten shows no threatening visage. They live, they seem to love, the Sun shines bright, they have eyes only for the things which Pleasure brings them, they hear the whispering winds and babbling brooks, and laugh in unison with the laughter of the Sea.

The daughter of Tethys and Chronos, the ever-virgin Goddess, the laughter-loving maid, sees them, she nods and smiles and promises to them an eternity of life and love, but as ever promises, false hearted that she is, only to deceive. Matrons forget their vows and maidens vow but falsely, and intoxicated by the environment, which they imagine that of "Freedom," they remember only that they are women, while they see in their companions only *Man* in all his multitudes of forms, but *never men.* Mentally and morally they find enjoyment without limit, and they take whatever gifts the gods provide for them, and very wise they ask no questions, "for when ignorance brings bliss 'tis but folly to be wise."

Yet this enchanting period ends, and the rush and hurry of the automobile, conveying those whose bank account will grant this mode of travel, or the plebeian express train hurries the wanderers homeward. They arrive,—and then? To the thoughtful physician who has for weeks been in the very middle of this strange and mysterious enchantment, this mystery of madness, the utter disregard of health, causes much uneasiness. He knows that at least seven out of every ten women have for days been living on their reserved strength, and he knows that reaction will set in the instant the strain is relaxed by the very least possible portion of the load, and he dreads lest an explosion be the next act in the drama.

Those who will be his patients begin presently to fall ill, and he who saw the beginning sees now the end of this mad campaign of pleasure. Ignoring enteric fever, and all the other forms of disease which can be diagnosticated as the work of individual mischief makers, the physician, who has been at the rendezvous of the pleasure seekers, at the corner in hill country, or at the seaside, where Dives and his wife and daughter have courted Hedone's good graces so recklessly, waits patiently for manifestations of the consequences of the midsummer-madness, and presently his patience is rewarded. He sees that Nemesis, as always, has walked unseen in her sister's shadow and that she exacts her penalty to the very last sigh, to the last fraction of a tear.

These women who returned in such splendid health, in such mad good spirits, are presently taken sick; no sooner have they returned, no sooner have they taken up the daily round of their duties and of their domestic lives, than something goes wrong and instantly they are invalids, not by the choice of any, and least of all by their own, but by compulsion. This illness is not the recoil from moral, mental and physical strain, as some have inferred, as has been said by many and taught by not a few, who are or were accounted great clinicians, and so accounted justly, nor is it an attack of "invalidism." On the contrary, in most cases the illness takes the form of a comparatively well known symptom picture, as a catalogued disease. At other times the effect produced is "malaise" only, or that state which a few years ago was everywhere and still is in some places held to be sufficiently described by the epithet, "an attack of malaria."

In considering the distressing condition, demanding our immediate aid, it is important to remember that certain real diseases, which are the result of well known causes, must be eliminated at once, as has already been referred to, such for example as the very common but protean "enteric fever," which is now well known to cause much more than half of that illness of the Autumn season for which we can account. This elimination is the more needful because but very little of the illness of this season is fully understood, and a great part of the printed traditions aids us not in the least degree by giving to us the much needed light; indeed, it rather hinders us by darkening counsels, and the verbal discussion which goes on around and about us appears to be little if at all more profitable, to wisdom of action or by advice.

One of the most interesting of these "end of the season" disorders is frequently mistaken for a condition so much more serious that the diagnostic blunder always causes extreme surprise, when the facts are all distinctly seen. Not that the condition in actual being is not sufficiently serious to demand the utmost care from the physician whose duty it may chance to be to care for the unfortunate; it is, but the commonly made diagnostic blunder is prone to cause a condition quite as serious as the real pathological condition; and when, as so often, all too often, happens, the original illness and the diagnostic blunder are both a burden thrown upon the mentality of the patient, it is no wonder that the mental burden becomes truly excessive.

The disorder begins to show its hydraheads almost at once, usually within a week or ten days of the return home. The patient, who to the very hour of return was active, full of laughter and joy, to all who come in contact with her appearing to be the very incarnation of high spirit, becomes listless, irritable, weary at trifles; and sometimes is given to outbursts of apparently inexplicable grief and causeless crying, and even at times to such passionate manifestations of emotion, at once ill defined and unexplained, as apparently to justify the suspicion of mental unsteadiness; and this suspected unsteadiness almost of a certainty appears prominently among the possible explanations which will presently be sought for with more eagerness than judgment, and with much more pertinacity than wisdom. A few such cases have been recorded during the past three years, principally in the French journals, which, if not embellished by the skilled imaginations of the story-tellers, certainly justify the conclusion that the most daring imagination approaches to facts only distantly.

It is said that a famous writer of fiction, having introduced a scene into the best known of his stories which turned on one of these causeless outbursts, a physician was so unwise as to say that this was "a very curious instance of the ignorance of laymen on matters connected with medicine." Thereupon the novelist referred him to the very page in a well known medical publication which contained the very same description, given as being an account of an actual happening. The writer also has been the witness of one of these outbursts of causeless, or apparently causeless, grief; and the utter tragedy of thought, word and gesture will never be forgotten. The real difficulty will always be to determine how much is due to "nervous storm" and how much to the occasion which has chanced to cause or call forth the terrifying outburst.

In some of theses cases, which afford symptoms suggesting troubles much worse than "serious," the unfortunate victim has been put in condition of restraint, and the actual facts have not been discovered until the mischief, done to the personality by this precaution, has been carried beyond any possibility of repair. Thus, in too large a group of cases, young women have been "put under observation" and have thus been indelibly stigmatised because the family physician first, and the consultant misled by him afterward, accepted as true a statement neither should have accepted without personal verification, and thus both failed, as they should not have done, in making a diagnosis at sight. More than one case of this kind has passed within my experience and the cases are not unique, nor even particularly uncommon, and at least two great specialists, one in England and one in the United States, have declared that the mistake is disgracefully frequent. The family physician is called in, maybe in the very midst of an impassioned outbreak, and a dreadful tale is told to him, and he, already impressed by the nervous unsteadiness of his victim and the condition of persistent mental excitement, asks for the privilege of consultation. It is of course granted and he, already convinced as to the facts, seeks the advice of a physician who is a specialist in nervous diseases.

The special practice of the consultant is concealed from the young woman by her parents, or those who act in their stead, but somehow the patient is informed of it, by her maid, usually, who imagines that by telling her mistress of the fact, which has been unwisely concealed by the physician, she is protecting her mistress from some plot against her peace. The fact of the concealment is thus brought to the notice of the victim, this unfortunate young woman, in such a way as to impress upon her already excited mind that those about her have become doubtful of her sanity, and that they believe that she is no longer sane. The suspicion reacts upon the imagination of the victimised young woman, and fear is added to the existing causes of excitement and mental perturbation, while the physical condition which underlies the apparent causes of disturbance gives ground for a very real despondency.

As might have been forseen, when the famous specialist is introduced to the young woman she presents every symptom of the suspected disorder of mind and yet not in such way as to convince the specialist beyond all hesitation. Yet the almost criminal, but worse than crime, for 'tis a blunder, mistake is made and the unfortunate victim is "placed under observation."

Thus is one more victim made, the unfortunate young woman is thus put under observation at the dictum of a man who has no real and legal right thus to pronounce sentence upon her, and yet he is wholly immune in respect to all responsibility for his act by reason of the way in which this act is done. The unfortunate is under observation, her doom is sealed; never in her life can this young woman be the same as other young women, she has been marked, one might say branded, as one of those unfortunates whose perfect sanity has been called in question; from this time onward for all her life she will be exposed to innuendo and suspicion, the innuendo and suspicion of her dearest friends, who will never hesitate to say, "Mollie is a delightful young lady, but very unfortunate. Do you know? No, well, she overdid one summer and it was necessary to send her to a sanitarium for observation." Naturally a sensitive young woman does not enjoy references so cruelly made, and she withdraws as much as possible from those who persistently disregard her feelings and almost intentionally cause her pain. Reaction naturally comes in time, but having learned the delights of solitude, she more and more inclines to enjoy its dangerous pleasures. Cut off from the companionship of those of her own age, partly by the acts of others and partly by her own, this unfortunate young woman is before long reduced to a selfish and largely fictitious valetudinarianism. She is not really ill and she knows it, but, as she finds the affectation of ill health less tedious than explanations, especially as the explanations do not explain, she accepts the situation passively. Gradually she yields more and more to the seductions of this life, until the affectation of valetudinarianism by necessity becomes her dominant idea, she believes that she is an invalid and magnifying every trifling pain into an agony, every insignificant headache into a menace to her sanity, even to her life, until the integrity of her sense of distinction and proportion is lost and she is reduced to *quasi* imbecility.

All of this miserable history is the direct result of the mistake in diagnosis, a mistake which should have been practically impossible, and a mistake which would have been easily avoided, both by the physician and the consultant, but for the physician's initial blunder in mistaking the accidental and secondary for the primary symptoms, the transient for the constant, the superficial for the fundamental. Had the family physician in seeking the solution of his problem, the establishment of his diagnosis, sufficiently difficult at best, without the added handicap of an expert opinion, much misled and more misleading, (that of the mother, sisters, friends, all ready to aid if possible, but with an opinion already formed to urge them to give special color to the atmosphere, and, having deceived themselves, to warp the physician's judgment also), the nervous system's disorder might have been assigned to its right place in the catalogue of symptoms, instead of being mistaken, so needlessly, for the phenomenon which should dominate the whole diagnostic field. In fact all that the mental group of the nervous system's manifestations really amounted to is simply an exhibit of certain interesting instabilities, an accidental detail—nothing more.

It is even possible that had the family physician asked, "What is the condition of the urine?" and followed up his question he would have saved the case. But it is quite certain that if he asked this question he did not follow up the inquiry as he should have done. It is even possible that a sample of the urine was sent to some laboratory for examination, yet were this done it is hardly likely that the chemist's report would aid much in clearing the diagnosis, because it would probably, almost certainly in fact, fail to mention—as probably the physician chemist made no note of it—the only fact of real importance to the diagnosis as to which the examination of the urine could afford any information, namely, the rapid oxidation of the pigment of the urine, by which the color is changed from a clear transparent yellow with a greenish opalescence to a dark, or even very dark, muddy brown. This simple fact, the change produced by the action of the air upon the pigmentary contents, would (or should) have given the physicians connected with the case a suspicion easily verified as to the possible causes of at least some of the phenomena observed,—the probable cause of most of them and the presumptive cause of all. In a word, had the physician carefully considered the facts in the way suggested, he could not but have noted that the urine contained the bile pigments in great quantity, which would at once have accounted to him for all the most prominent symptoms, which, obviously, after the observed discovery of the bile pigment in quantity, are simply phenomena to be expected, and present, proclaim the diagnosis in the tones of Stentor.

Bilious intoxication is not so unusual as to cause any special alarm to the experienced physician, and that bilious intoxication is the condition which he has to deal with the experienced practitioner will plainly recognise when once the facts in relation to the urine pigment are made plain. But it must be remembered that it is only by some accident, or the physician's thoughtfulness, that these facts can be learned in houses provided with those various appliances for personal comfort and cleanliness, which were luxuries among even the well placed two generations since, but which are regular necessities of life today.

Having determined the fact of the biliary poisoning, the next problem is the reason of it, why it is and wherefore, and it is by no means always an easy thing to establish the answers to these questions, for the patient will almost to a certainty insist that the bowels are fully emptied every day. However this may be, an examination of the abdomen by palpation will demonstrate that the large intestine contains much refuse, which should be removed at once, using such speed as may seem to the physician to be possible, or wise.

The half, or even the quarter, of a century since, this condition, if the physician was so fortunate as to recognise the significance of the symptoms, would have been looked upon, and rightly too, as a subject for much thought and consideration, but today, thanks to the extended studies of Bouchard and those who, inspired by the wonderful results which he obtained, followed his steps and then outstripped him, this question in therapeutics is much simplified, and even in some ways reduced to a matter of routine. The attending physician who has been sufficiently acute to establish the diagnosis, we may be assured is sufficiently informed as to the therapeutic possibilities to meet the indications of the condition which he has surprised. Naturally, adopting modern ideas, means and methods, he acts at once and without hesitation. To relieve at once the condition which displeases him he empties the bowel speedily, cleanses it effectually, disinfects it carefully, and having thus removed the cause which originated the threatened danger, he guards against the possibility of its renewal. Guided by a well systematised opinion, his experience directs what to do, he hastens slowly, acts rapidly, succeeds at once. Only two questions demand anxious thought. "Shall I empty the gut by a single operation or by degrees?" and "has this constipation continued so long as to render the over distension of the rectum with hard masses possible?"

Forty years ago, or even twenty-five, the physician would probably have made use of drugs tending to excite violent peristalsis even if he had also used a saline; and these drugs by causing such peristalsis would also cause a discharge of serum into the lumen of the gut, thus developing a seromucus lubricating fluid, which, to quiet an objecting intelligence, he assured himself was also depurative, although being intelligent he doubted it. Today the physcian gives the saline and he gives it alone, and in giving it he seeks the solution of therapeutic problems which his father understood but dimly, and of whose existence his grandfather did not even dream. Among salines, he has much liberty of choice, and therefore the duty of selection; this his grandfather had not at all and his father only in small measure, while today the physician has the full duty of determining "which one?" among at least a dozen, and must give a reason for his decision to a presumably rather exacting scientific conscience. His grandfather knew only the neutral sulphate and the neutral and acid tartrates, and he knew of no reason for a selection among them except his own personal taste and choice. His father's list contained, in addition to these, the effervescing mixture of the acid tartrate of potash with the neutral, or better the acid, carbonate of an alkali, and this was widely used by all.

But the physician today has a wider choice and a greater responsibility for he has to consider two distinct types of saline, the still and the effervescing salines, and in this second type list are two distinct kinds which may properly enough be called two species, the rapidly effervescing and the slowly effervescing salines, neutral salines, that is. In those of the first type the carbondioxide causing the compound to foam is given off so rapidly that practically none of its gaseous contents can reach the stomach, and surely little of it can by any chance extend its beneficent influence beyond the pylorus. Not so the others, disengaging the carbondioxide gas but slowly; a very large fraction of this component is liberated in the small intestine to become useful in a way frequently not thought of at all, and very seldom considered with that seriousness which is the due of so useful, and it may be even powerful, an agent, powerful as measured in the effects produced in comparison with the reaction of the system, which reaction is far less than the observing physician might expect.

When the solution of crystalloid salts reaches the obstructing mass in the lumen of the intestine it can go no farther, and it reaches this position in from three to five minutes after it is taken into the stomach, commonly sweeping through this viscus as if the small intestine were not dilated at this point. Thus, the water ingested washes out and carries before it all and singular everything it has met with in the upper or small intestine.

On its way from the pylorus to the ileocecal valve the saline solution has been steadily disengaging carbondioxide, until the bulk of the intestine has become twice or even thrice the bulk of the ingested fluid, and in this journey the water has been in advance of the gaseous portion taking up the various mucoid and albuminous elements until its fluidity is much reduced, sometimes to an index as small as N|3. In passing into the ascending colon the water goes first, but in the ascending colon positions change and the gas coming to the top forms an elastic cushion partly or wholly gaseous, partly water mixed, suds like in condition, containing vast numbers of small bubbles, which mixture of gas and water makes its way into the various material substances which as a mass plugs the intestine, and it does this much more rapidly than water alone, or water mixed with mucus but without the gaseous content possibly could do it. The water under the action of the peristalsis presses upon the gaseous layer crowding this into the interstices of the refuse contained in the gut, breaks this up and gives opportunity for the water to enter the crevices, and in this way the water and gas together rapidly disintegrate that which would long have resisted the action of water alone. At the same time the fecal masses and water are rapidly churned into a practically homogeneous fluid by the action of the peristalsis in the ascending and the transverse colon. In the descending colon the action of the water in disintegrating the contents is less rapid until the sigmoid flexure is reached, when the action again increases in rapidity. This because the sigmoid flexure is converted into a double funnel, with the large ends contiguous, as soon as peristalsis is excited therein, the descending colon and the straight gut entering and leaving it at right angles nearly. The large impeding mass which is the result of every twenty-four hours' sluggishness is thus disintegrated and broken up and mixed with gas and water so completely that in a very short time, both actually and relatively, emptiness and the consecutive reaction against the distended conditions are easily, surely and without discomfort secured to the patient, a result which could not be obtained with so little discomfort by any cathartic drug, nor so rapidly by any saline not disengaging carbondioxide after it had been taken into the intestine proper, that is, had passed the pyloric sphincter.

It is asserted by an observer who has had an abundant opportunity for observation, that we may compare the rapidity of the action of aloes, epsom salt and the rapidly effervescing and slowly effervescing salines, and that the reaction is as the continued proportion, 1; 4; 5; 9, the reaction to the rapidly effervescing saline solution being hardly more rapid than the reaction to the still saline solution, while the reaction to the slowly effervescing saline solution is twice the mean between the rapidly effervescing and the still solution. This is the scientific statement of the reason why of the phenomenon I made note of in a paper published some months ago, that the Abbott effervescing saline which gives off carbondioxide slowly and for a considerable period, but gradually parting with its gaseous contents, not giving it up with almost explosive rapidity so as to foam "all over everything" as some effervescing salines do, was much the most efficient agent among the members of this group in relieving the condition commonly called sluggishness of the intestines or chronic constipation, both of which unfortunate phrases are used for this condition, and for it both alike are misleading appellatives.

But to return to the patient with whose case we began and from which we have so far wandered away, had the attending physician just happened to remember that his patient had been taking violent exercise from morning until night during a period of about sixty days, and that during these sixty days she had been eating of food adroitly prepared by skilled cooks, in every dish of which was hidden from sight, but functionating actively, one or more liver stimulating, catharsis producing drugs; and that his patient during the same period had been taking active exercise. I say, had he called all this to mind and then compared the diet to which she had been subjected since her return and the complete absence of all exercise but the very minimum with which the life's functions could have being, had he remembered these two conditions, and the other various facts which he should have seen without any serious effort, would he not, I ask it in good faith, have made the diagnosis without calling the young woman's sanity into question and sending her to a private hospital for observation?

Granted that the physicians at the sanitarium had a better opportunity for making a correct diagnosis, they took much more trouble to secure this correct diagnosis than the family physician did, but was it not an obligation laid upon him to take just this same amount of trouble and to make just as accurate a diagnosis as did the sanitarium physician, before he put a black mark upon the mental reputability of any one, and more especially, of one who could not defend herself against an intangible foe, against the suspicions of her dearest friends?

If physicians would make it a practice in all these cases to wait twenty-four hours, or even twice or thrice as long before they consent to give a diagnosis, and meanwhile would carefully and systematically empty the intestinal tract, and make proper urinary examinations before or after so doing, would not more of these cases meet with a satisfactory and speedy elucidation? An elucidation obtained without sending the patient to a sanitarium for observation as to the mental state, and the consequent innuendo in respect to their sanity, the perfect balance of their minds.

THE SINCLAIR, 163 FULTON ST.

